# Draft Genome Sequence of Aspergillus tubingensis WU-2223L, a Citric Acid-Producing Filamentous Fungus Belonging to *Aspergillus* Section *Nigri*

**DOI:** 10.1128/MRA.00702-20

**Published:** 2020-08-13

**Authors:** Isato Yoshioka, Hiroki Takahashi, Yoko Kusuya, Takashi Yaguchi, Kohtaro Kirimura

**Affiliations:** a Faculty of Science and Engineering, Waseda University, Tokyo, Japan; b Medical Mycology Research Center, Chiba University, Chiba, Japan; c Molecular Chirality Research Center, Chiba University, Chiba, Japan; d Plant Molecular Science Center, Chiba University, Chiba, Japan; Broad Institute

## Abstract

Aspergillus tubingensis WU-2223L, belonging to the section *Nigri*, is a hyperproducer of citric acid. Here, we present the high-quality draft (35 Mb) and mitochondrial (32.4 kb) genome sequences of this strain, which consisted of 16 scaffolds in total. The draft and mitochondrial genome sequences comprised 11,493 and 15 genes, respectively.

## ANNOUNCEMENT

Citric acid (CA) is industrially produced by fermentation using the filamentous fungus Aspergillus niger (1). Strain WU-2223L, isolated in 1960 from Japanese soil and identified as A. niger (2), was reidentified as A. tubingensis based on recent phylogenetic classification (3). Since strain WU-2223L produces large amounts of CA (>50% [wt/wt] from supplied glucose) (2, 4, 5), the genome sequence of A. tubingensis WU-2223L will enhance our understanding of the mechanisms underlying CA hyperproduction.

A. tubingensis WU-2223L, deposited as NBRC111403 in the National Institute of Technology and Evaluation Biological Resource Center and as IFM 65578 in the Medical Mycology Research Center, Chiba University (through the National BioResource Project [NBRP]), was used in this study. The genome sequence was produced using a hybrid assembly approach of Illumina and Oxford Nanopore Technologies (ONT; Cambridge, UK). The genomic DNA of A. tubingensis WU-2223L was extracted from mycelia cultivated in potato dextrose broth at 25°C for 1 day using phenol-chloroform extraction and a Genomic-tip 20/G (Qiagen, Hilden, Germany). A DNA library for Illumina sequencing was prepared using a NEBNext Ultra II FS DNA library prep kit for Illumina (New England Biolabs, Ipswich, MA). Paired-end (PE) sequencing of 2 × 150-bp reads was performed by BGI (Kobe, Japan) on a HiSeq X platform (Illumina, San Diego, CA). A DNA library for ONT sequencing was prepared using a short-read eliminator kit (Nippon Genetics, Tokyo, Japan) and a ligation sequencing kit (SQK-LSK109; ONT), and sequencing was performed on a MinION platform (ONT) with a FLO-MIN106D flow cell (R9.4.1; ONT) for 48 h according to the manufacturer’s instructions. A total of 474,042 ONT reads (8.7 Gbp) were generated with a mean length of 18,464 bp using ONT Guppy version 2.3.5 and subsequently filtered using Filtlong version 0.2.0 (https://github.com/rrwick/Filtlong) with Illumina PE reads. The subsampled ONT reads were corrected using Canu version 1.8 (6). The parameter settings for software are shown in Table 1.

**TABLE 1:** Parameter settings used for genome sequencing and assembly

Software	Parameters
Guppy	Options --qscore_filtering, --flowcell, FLO-MIN106, --kit SQK-LSK109, --enable_trimming, true, and --cpu_threads_per_caller 10
Filtlong	Options --min_length 100, --keep_percent 90, --target_bases 4000000000, --trim, and --split
Canu	Options nanopore-raw and genomeSize=37m
BWA	Default
Pilon	Default
MaSuRCA	Options FLYE_ASSEMBLY=1 and LHE_COVERAGE=25

The draft genome of A. tubingensis WU-2223L was assembled using MaSuRCA version 3.3.3 (7) with 19,102,449 PE reads (162× coverage) and 32,827 of the corrected ONT reads (38× coverage; 1.3 Gbp) and was iteratively polished four times using Burrows-Wheeler Aligner (BWA) version 0.7.17 (8) and Pilon version 1.23 (9). A total of 16 scaffolds was obtained, and a total draft genome size of 35,047,259 bp (49.32% G+C content) was determined with that of a mitochondrial genome. The *N*_50_ value and the maximum scaffold size were 3,153,776 bp and 5,352,872 bp, respectively. A total of 11,493 protein-coding genes were predicted using FunGAP version 1.0.1 (10) with transcriptome reads of A. tubingensis CBS 134.48 (GenBank accession number PRJNA358285) and an Aspergillus oryzae gene model for AUGUSTUS (11) inside FunGAP. Totals of 270 tRNAs and 68 rRNAs were predicted using tRNAscan-SE version 1.3.1 (12) and RNAmmer version 1.2 (13), respectively. For the mitochondrial genome with 32,393 bp, 15 open reading frames (ORFs) and 26 tRNAs were predicted using GeSeq (14) and tRNAscan-SE, respectively.

### Data availability.

The whole and mitochondrial genome sequences of A. tubingensis WU-2223L were deposited at DDBJ/EMBL/GenBank under the accession numbers BLWE01000001.1 to BLWE01000015.1 and LC545447.1, respectively. The raw sequencing reads were submitted to the Sequence Read Archive under the accession number DRA010281.
